# Multimodal ultrasound in the assessment of muscle involvement in systemic sclerosis patients: results from a bicentric study

**DOI:** 10.1093/rheumatology/keaf415

**Published:** 2025-08-06

**Authors:** Riccardo Bixio, Stefano Di Donato, Maria Giovanna Lommano, Gianluca Smerilli, Sonia Farah, Seda Çolak, Marco Minerba, Francesca Pistillo, Richard J Wakefield, Fausto Salaffi, Ombretta Viapiana, Rossella De Angelis, Maurizio Rossini, Edoardo Cipolletta, Emilio Filippucci, Luca Idolazzi, Paul Emery, Francesco Del Galdo, Andrea Di Matteo

**Affiliations:** Rheumatology Unit, University of Verona, Verona, Italy; Faculty of Medicine and Health, Leeds Institute of Rheumatic and Musculoskeletal Medicine, University of Leeds, Leeds, UK; Faculty of Medicine and Health, Leeds Institute of Rheumatic and Musculoskeletal Medicine, University of Leeds, Leeds, UK; Rheumatology Unit, Dipartimento di Scienze Cliniche e Molecolari, Università Politecnica delle Marche, Jesi, Ancona, Italy; Rheumatology Unit, Dipartimento di Scienze Cliniche e Molecolari, Università Politecnica delle Marche, Jesi, Ancona, Italy; Rheumatology Unit, Dipartimento di Scienze Cliniche e Molecolari, Università Politecnica delle Marche, Jesi, Ancona, Italy; Faculty of Medicine and Health, Leeds Institute of Rheumatic and Musculoskeletal Medicine, University of Leeds, Leeds, UK; Department of Rheumatology, Gülhane Training and Research Hospital, University of Health Sciences, Ankara, Turkiye; Faculty of Medicine and Health, Leeds Institute of Rheumatic and Musculoskeletal Medicine, University of Leeds, Leeds, UK; Rheumatology Unit, University of Verona, Verona, Italy; Faculty of Medicine and Health, Leeds Institute of Rheumatic and Musculoskeletal Medicine, University of Leeds, Leeds, UK; Rheumatology Unit, Dipartimento di Scienze Cliniche e Molecolari, Università Politecnica delle Marche, Jesi, Ancona, Italy; Rheumatology Unit, University of Verona, Verona, Italy; Rheumatology Unit, Dipartimento di Scienze Cliniche e Molecolari, Università Politecnica delle Marche, Jesi, Ancona, Italy; Rheumatology Unit, University of Verona, Verona, Italy; Rheumatology Unit, Dipartimento di Scienze Cliniche e Molecolari, Università Politecnica delle Marche, Jesi, Ancona, Italy; Academic Rheumatology, University of Nottingham, Nottingham, UK; Rheumatology Unit, Dipartimento di Scienze Cliniche e Molecolari, Università Politecnica delle Marche, Jesi, Ancona, Italy; Rheumatology Unit, University of Verona, Verona, Italy; Faculty of Medicine and Health, Leeds Institute of Rheumatic and Musculoskeletal Medicine, University of Leeds, Leeds, UK; Faculty of Medicine and Health, Leeds Institute of Rheumatic and Musculoskeletal Medicine, University of Leeds, Leeds, UK; Faculty of Medicine and Health, Leeds Institute of Rheumatic and Musculoskeletal Medicine, University of Leeds, Leeds, UK; Rheumatology Unit, Dipartimento di Scienze Cliniche e Molecolari, Università Politecnica delle Marche, Jesi, Ancona, Italy

**Keywords:** muscle, ultrasound, systemic sclerosis, quality, mass, stiffness, shear-wave elastography, muscle strength, physical performance, disability

## Abstract

**Objective:**

To investigate muscle mass, quality, and stiffness using ultrasound (‘multimodal ultrasound’) in systemic sclerosis (SSc) patients, compared with healthy controls (HCs), and examine their correlation with muscle strength, physical performance and disability.

**Methods:**

In this cross-sectional, bicentric study (Jesi and Leeds), ultrasound scans of the quadriceps muscle (QM) were performed in SSc patients (without inflammatory myositis) and HCs to assess muscle mass, quality [using a semi-quantitative modified Heckmatt scale (mHS) and grey-scale histogram analysis (GSA) for muscle echogenicity], and stiffness [measured by shear-wave elastography (SWE)]. Muscle strength was assessed using the handgrip test, physical performance was evaluated with the Short Physical Performance Battery, and disability was measured using the Health Assessment Questionnaire.

**Results:**

A total of 81 SSc patients (36 from Jesi, 45 from Leeds) and 24 HCs (Jesi) were included. After adjusting for age, SSc patients showed increased muscle echogenicity—measured by mHS and GSA (the latter in the Jesi cohort; *P* < 0.001)—and lower SWE values (Jesi cohort, *P* < 0.001), with no significant difference in muscle mass (*P* = 0.433). Higher QM muscle thickness values significantly correlated with better strength, better physical performance and lower disability. Increased muscle echogenicity (i.e. low muscle quality) significantly correlated with poorer strength, worse physical performance and higher disability. Higher SWE values in the Jesi cohort significantly correlated with better strength, better performance and lower disability, while 2D SWE in the Leeds cohort showed reduced strength but no link to disability.

**Conclusion:**

Ultrasound demonstrated its potential for detecting early, clinically significant changes in muscle mass, quality and stiffness in SSc patients.

Rheumatology key messages

- Multimodal ultrasound assesses muscle changes in patients with systemic sclerosis.
- Systemic sclerosis patients show reduced muscle quality and altered stiffness on ultrasound.
- Muscle ultrasound findings in systemic sclerosis correlate with weakness and functional impairment.

## Introduction

Systemic sclerosis (SSc) is an autoimmune disease characterized by microvascular changes, fibrosis and inflammation affecting multiple organs, especially the skin, lungs and gastrointestinal tract (GIT) [1].

Muscle involvement is common in SSc, with contributing factors including reduced activity, muscle and systemic inflammation, drug use (e.g. steroids), and nutritional deficiencies [2, 3].

Sarcopenia, defined as the loss of muscle mass, strength and function, affects up to 22% of SSc patients and is linked to reduced quality of life, functional decline and increased mortality, particularly in those with SSc-associated interstitial lung disease [2, 4, 5]. Given these complications, early detection of muscle involvement in SSc patients is crucial for effective management. In addition, early interventions, such as regular exercise, medication or supplements, may help prevent or mitigate muscle disease progression and improve patient outcomes [6].

Imaging plays a relevant role in assessing reduced muscle mass and quality, two of the three key criteria for sarcopenia-related muscle involvement, with the third criterion being reduced physical performance [7]. Dual-energy X-ray absorptiometry (DXA) is the gold standard for muscle mass assessment but lacks muscle quality data and involves radiation [8]. Magnetic resonance imaging (MRI) excels in detecting inflammatory myopathy but is costly, time-consuming and impractical for routine sarcopenia screening [9].

Ultrasound (US) is a promising, cost-effective, real-time and non-invasive tool for assessing sarcopenia in both elderly individuals (primary sarcopenia) and patients with rheumatic diseases (secondary sarcopenia) [10]. It detects muscle atrophy and qualitative changes like increased echogenicity, indicating fibrosis or fat infiltration [11]. US shear-wave elastography (SWE) is also emerging as a method for evaluating muscle stiffness, offering insights into muscle physiology and biomechanics [12].

A previous study from our group found that systemic lupus erythematosus (SLE) patients without overt sarcopenia or myositis exhibit early muscle quality and stiffness changes detectable by US [13]. These changes correlated with reduced key clinical measures of sarcopenia, such as grip strength and physical function, underscoring the potential of US for identifying clinically relevant muscle alterations in SLE and, potentially, in other rheumatic conditions.

In SSc patients, sarcopenia-related muscle involvement remains largely underexplored, with only one study to date assessing muscle mass using US. Notably, muscle quality and stiffness have been scarcely investigated in SSc patients using US, representing a significant gap in the literature [3, 14, 15].

The main objectives of the current study were (i) to investigate the US findings related to muscle mass, muscle quality and muscle stiffness (‘multimodal US’) in patients with SSc compared with healthy controls (HCs), and (ii) to examine in patients with SSc the correlations between various US muscle assessment methods and their association with muscle strength, physical performance and disability.

Additional objectives included evaluating the correlations between different US muscle assessment methods (i.e. muscle mass, quality and stiffness) in SSc patients and assessing the relationships between US findings and both demographic and clinical characteristics in these patients.

## Methods

### Study design and patients

This cross-sectional study included consecutive SSc patients from two tertiary rheumatology centers: Jesi, Italy (August–December 2021), and Leeds, UK (April–June 2024). Participants were ≥18 years old and met the 2013 American College of Rheumatology/European Alliance of Associations for Rheumatology SSc criteria [16].

Exclusion criteria included motor disability, walking aid use, recent major surgery (<3 months), and inability to perform the handgrip (HG) test. Other exclusion criteria were known malignancy, active inflammatory myositis (elevated creatine kinase or MRI/nerve conduction abnormalities), and malnutrition (BMI <18.5 kg/m^2^ or >10% weight loss in 6 months). HCs were recruited from Jesi’s ‘Carlo Urbani’ Hospital staff (i.e. staff members and their relatives or friends), applying the same exclusion criteria.

### Population characteristics

In SSc patients, collected data included age, sex, BMI, smoking status, disease duration and autoantibody profiles. Nailfold videocapillaroscopy was performed to assess capillaroscopic patterns [17]. Disease manifestations recorded included Raynaud’s phenomenon, history of digital ulcers (DUs), sclerodactyly, skin fibrosis assessed by the modified Rodnan skin score (mRSS), and interstitial lung disease confirmed by high-resolution computed tomography. Pulmonary arterial hypertension was diagnosed via right heart catheterization. Articular involvement was defined as joint synovitis (i.e. joint pain and swelling) [18], while cardiac involvement included left ventricular ejection fraction <45%, pericardial effusion or arrhythmia. GIT included the presence of severe gastroesophageal reflux disease requiring chronic proton pump inhibitor therapy, dysphagia, early satiety, unintentional weight loss, confirmed small intestinal bacterial overgrowth, or ≥1 episode of malnutrition requiring parenteral nutrition. Dyspnoea severity was assessed using the modified Borg scale [19]. The SSc phenotype was classified as limited cutaneous SSc or diffuse cutaneous SSc [20]. Scleroderma renal crisis history was documented, and treatment regimens were recorded. For HCs, collected data included age, sex, smoking status, and BMI.

### Muscle strength

All SSc patients and HCs underwent a HG test for the evaluation of muscle strength. In the Jesi cohort, a cylindrically shaped device made of five force sensors (FSR-402) (Interlink Electronics, Irvine, CA, USA, connected to an Arduino Mega 2560, Monza, Italy) was used [21]. In the Leeds cohort, the Jamar Hydraulic Hand Dynamometer (Jamar, Patterson Medical, Warrenville, IL, USA) was used [22]. During the HG test, participants sat with the elbow at 90°, forearm neutral, and wrist extended 0–30°. The dynamometer was maximally squeezed for 3 s, performing three trials per hand with 1-min rests. The highest value was recorded. The European Working Group on Sarcopenia (EWGSOP2) criteria, which defines patients with HG test <27 kg (if male) or <16 kg (if female), were used to define low muscle strength and ‘probable sarcopenia’ [8].

### Physical performance and disability

In the Jesi cohort, all SSc patients and HCs completed the Short Physical Performance Battery (SPPB), a validated tool for assessing physical performance (balance, gait speed and lower limb strength). Each component is scored from 0 (poor) to 4 (best), with a total score ranging from 0 to 12, where higher scores indicate better function [23]. We defined a score ≤8 as indicative of reduced physical performance, per the EWGSOP2 guidelines [8]. The SPBB was not available for the Leeds cohort. Functional disability in SSc patients and HCs was assessed using the Health Assessment Questionnaire (HAQ), which covers eight daily activity domains. Scores range from 0 to 3, with higher scores indicating greater disability [24].

### Ultrasound assessment

US assessments were performed on all SSc patients and HCs by two experienced operators (A.D.M. and R.B., with 13 and 6 years of musculoskeletal US experience, respectively), both blinded to participants’ clinical and demographic data.

During the examination, subjects lay in a supine, neutral position with legs extended. In the Jesi cohort, US was performed using a MyLab X9 (Esaote SpA, Genova, Italy) with a 3–11 MHz broadband linear probe. In the Leeds cohort, an Aixplorer system (SuperSonic Imagine, Aix-en-Provence, France) with a SuperLinear™ SL10-2 MHz probe was used. Both systems used the same US settings: frequency 9.0 MHz, gain 50 dB and depth 5 cm (6 cm in obese patients where the femur surface was not visible).

Transverse scans of the quadriceps muscle (QM) were acquired at the midpoint between the anterior superior iliac spine and the upper pole of the patella, as described previously [13].

Muscle thickness was calculated bilaterally by summing the thickness of the rectus femoris (RF) and vastus intermedius (VI) muscles.

Muscle echogenicity was evaluated using two methods: a semiquantitative scale (a modified Heckmatt scale, mHS) recently developed by the authors, and grey-scale histogram analysis (GSA) using ImageJ software [25, 26], as previously described [13]. Region of interests (ROIs) were set within the RF and VI muscles, excluding fascia and bone. ImageJ (version 1.53e) calculated mean grey-scale intensity values from 0 (black) to 255 (white). GSA analyses were performed separately for each cohort by blinded assessors (S.F. and S.C.). Due to the use of different US systems at the two study sites, and the known influence of machine-specific settings, system gain and backend processing algorithms on grey-scale image intensity [27, 28], GSA was analysed separately for each cohort. Supplementary Fig. S1 illustrates different pictures of increased muscle echogenicity observed in patients with SSc.

**Figure 1.**
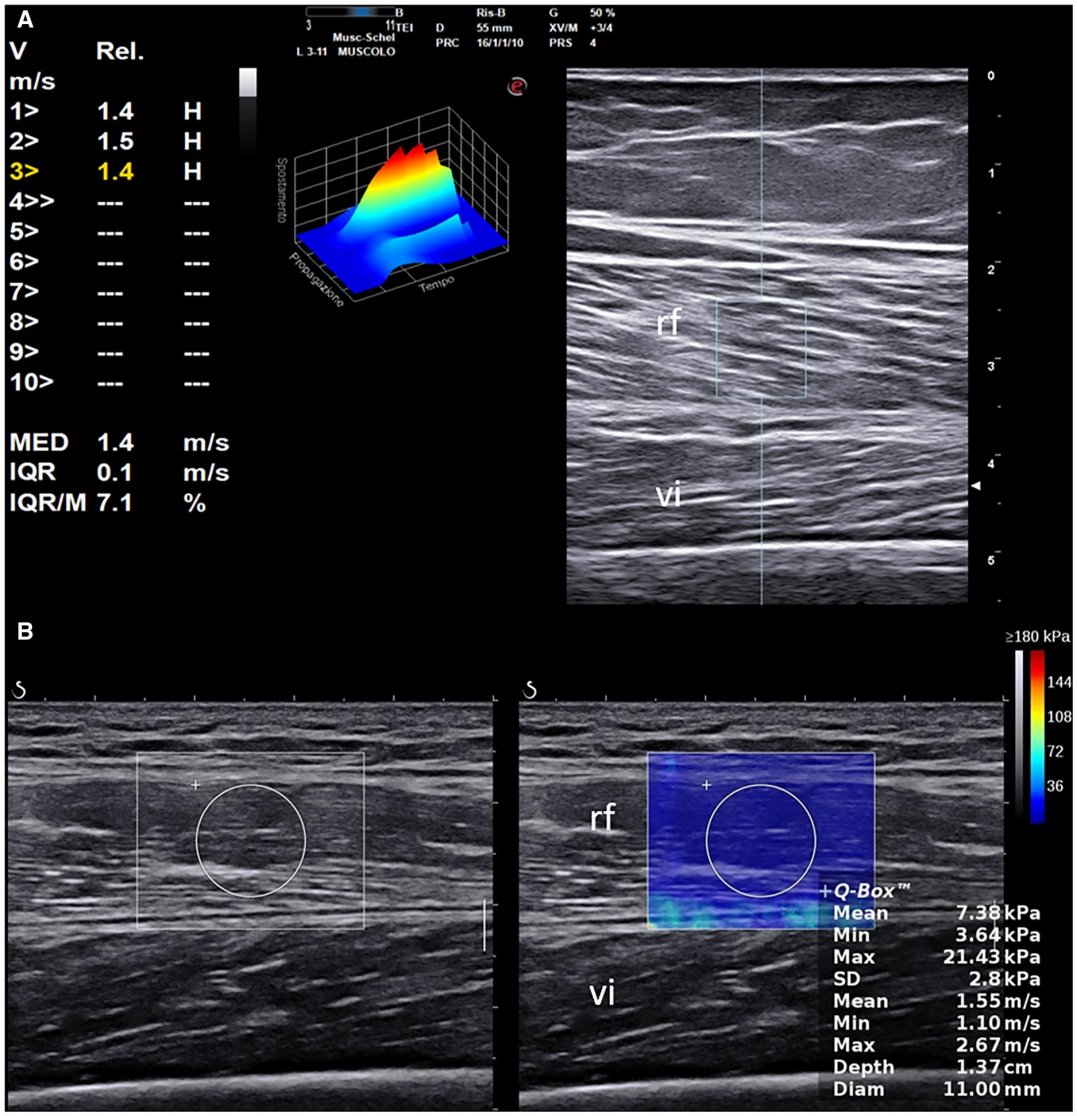
Shear-wave elastography measurements in SSc patients. Ultrasound images of the rectus femoris muscle in two patients with systemic sclerosis (SSc). (A) Point shear-wave elastography (pSWE) acquisition in a patient with diffusely increased muscle echogenicity. (B) 2D shear-wave elastography (2D SWE) in a different patient, with mildly reduced muscle thickness and increased echogenicity, particularly in the lower rectus femoris. Measurement regions are indicated: a rectangle for pSWE and a rounded area within a rectangle for 2D SWE. Results are in metres per second. Labels include rf (rectus femoris) and vi (vastus intermedius)

SWE was also conducted at the QM using a longitudinal probe orientation along RF muscle fibres, ensuring minimal external pressure. Four scans were performed on each leg using standardized anatomical landmarks [13]: two at the midpoint (1 cm medial and lateral to the central aponeurosis) and two 2 cm proximally. In Jesi, point SWE (pSWE) was used (Fig. 1A); in Leeds, 2D SWE (Fig. 1B) was employed. In each scan, a ROI was placed centrally in the RF, avoiding fascia, and each area was measured three times. The median SWE velocity (m/s) and interquartile range (IQR) were recorded.

Due to methodological differences and the lack of validated comparability between pSWE and 2D SWE—particularly given prior studies showing significant discrepancies in liver stiffness measurements [29, 30]—SWE results were analysed separately for each cohort. As HCs were only recruited in the Jesi cohort, direct comparisons with SSc patients from the Leeds cohort (who underwent GSA and 2D SWE) were not feasible. Nonetheless, GSA and SWE data from the Leeds cohort were included in analyses exploring associations between US muscle parameters (mass, quality and stiffness) and clinical features such as muscle strength and function in SSc patients.

### Statistical analysis

Normality was assessed graphically and using the Kolmogorov–Smirnov test (*P* < 0.05 indicating non-normal distribution). Continuous variables were reported as mean (s.d.) or median (IQR), while categorical variables were expressed as totals and proportions. Group differences were analysed using Student’s *t-*test or Mann–Whitney *U*-test for continuous data and chi-squared or Fisher’s exact test for categorical data. Comparisons between SSc patients and HCs included age, BMI, smoking status, HG test, HAQ, SPPB and US measures. Correlations among US measures were analysed using Pearson’s or Spearman’s coefficients. ANCOVA and Quade’s ANCOVA were adjusted for age in non-matched groups. The *q*-value (<0.05) controlled the false discovery rate. Analyses were conducted using R (v4.3.2; R Foundation for Statistical Computing, Vienna, Austria) and RStudio (PBC, Boston, MA, USA).

### Ethical approval

This study was approved by the local ethics committee for the Jesi cohort (CERM no. 155/2021) and the NHS Health Research Authority (Research Ethics Committee reference 15/NE/0211) for the Leeds cohort. All individuals participating in the study provided written informed consent.

## Results

### Population characteristics

A total of 81 SSc patients (36 Jesi cohort; 45 Leeds cohort) and 24 HCs (Jesi cohort) were included in the current study. The main clinical and demographic characteristics of the SSc patients and HCs are presented in Table 1, while the SSc disease-specific characteristics are reported in Supplementary Table S1. Overall, SSc patients were significantly older compared with HCs. The BMI was significantly higher in SSc patients than HCs, but statistical significance was not retained after correction for multiple comparisons.

**Table 1.** Demographics and clinical characteristics of SSc patients and healthy controls

Characteristic	SSc patients (*n* = 81)	Healthy controls (*n* = 24)	*P*-value	***q*-value** ^b^
Age, median (IQR), years	58 (52, 67)	45 (39, 54)	<0.001	<0.001
Male sex, *n* (%)	11 (13.6)	2 (8.3)	0.7	>0.9
Disease duration, median (IQR), years	7 (3, 11)			
BMI, median (IQR), kg/m^2^	24.6 (21.8, 29.3)	22.5 (20.6, 24.7)	0.050	0.10
Smoker, *n* (%)	6 (7.8)	2 (8.3)	0.2	0.3
HAQ, median (IQR)	0.50 (0.25, 1.13)	0.00 (0.00, 0.00)	0.2	0.3
Handgrip, mean (s.d.), kg	27.4 (9.6)	34.2 (9.6)	0.003	0.003
SPPB^a^, median (IQR)	10.5 (10.25, 12)	12 (12, 12)	0.002	0.003

a Measured in the Jesi cohort.

b False discovery rate adjustment for multiple comparisons. HAQ: health assessment questionnaire; SPPB: short physical performance battery; SSc: systemic sclerosis.

As shown in Supplementary Table S2, the Leeds cohort had higher HAQ scores and lower HG test values, while the Jesi cohort had a higher median mRSS and more anti-topoisomerase I antibody (ATA) positive cases. Leeds also had a higher prevalence of DUs and GIT involvement. Treatment-wise, Jesi patients used more endothelin receptor antagonists, and Leeds had more patients on phosphodiesterase type 5 inhibitors.

### US muscle findings

As shown in Tables 2 and 3, SSc patients had significantly lower QM thickness, increased muscle echogenicity—measured by mHS and GSA (the latter in the Jesi cohort; *P* < 0.001)—and lower muscle stiffness (pSWE, Jesi cohort) compared with HCs. After performing ANCOVA to account for age differences between the two groups, muscle mass was no longer significantly lower in SSc patients compared with HCs. By contrast, muscle echogenicity (both mHS and GSA) and pSWE values remained significantly different in SSc patients and HCs.

**Table 2.** US muscle assessment (muscle mass and quality) in SSc patients and healthy controls across the Jesi and Leeds cohorts

Characteristic	SSc patients (*n* = 81)	Healthy controls (*n* = 24)	*P*-value	***q*-value** ^a^
Muscle mass, mean (s.d.), mm				
Left				
VI muscle	12.32 (4.06)	14.85 (4.62)	0.011	
RF muscle	14.13 (4.59)	16.04 (4.18)	0.071	
QM muscle	26.49 (7.53)	30.89 (7.83)	0.014	
Right				
VI muscle	12.05 (3.94)	15.35 (4.62)	0.001	
RF muscle	14.6 (4.67)	16.22 (3.85)	0.124	
QM muscle	26.87 (7.85)	31.59 (7.44)	0.01	
Bilateral				
VI muscle	12.19 (3.78)	15.1 (4.53)	0.002	0.119
RF muscle	14.37 (7.37)	16.13 (3.92)	0.077	0.987
QM muscle	26.68 (7.37)	31.24 (7.55)	0.009	0.433
Echogenicity, median (IQR)				
mHS (left)	2.00 (1.00, 2.00)	0.00 (0.00, 1.00)	<0.001	
mHS (right)	2.00 (1.00, 2.00)	0.00 (0.00, 0.25)	<0.001	
mHS (bilateral)	1.50 (1.00, 2.50)	0.00 (0.00, 0.50)	<0.001	<0.001

a Adjusted by age (adjustments are performed only for bilateral measurements as more reflective of the patients’ global features). HCs: healthy controls; IQR: interquartile range; mHS: modified Heckmatt scale; QM: quadriceps muscle; RF: rectus femoris muscle; SSc: systemic sclerosis; US: ultrasound; VI: vastus intermedius muscle.

**Table 3.** Comparison of GSA and pSWE values between SSc patients and healthy controls (Jesi cohort)

Characteristic	SSc patients (*n* = 36)	Healthy controls (*n* = 24)	*P*-value	***q*-value** ^a^
Echogenicity, median (IQR), mean pixel intensity				
GSA (left)	96 (88, 104)	74 (56, 83)	<0.001	
GSA (right)	88 (83.3, 98.3)	70 (51, 82)	<0.001	
GSA (bilateral)	93.3 (86.7, 100.9)	71 (54, 82)	<0.001	<0.001
Muscle stiffness, median (IQR), m/s				
pSWE (left)	1.29 (1.15, 1.42)	1.69 (1.57, 1.83)	<0.001	
pSWE (right)	1.3 (1.2, 1.47)	1.68 (1.60, 1.90)	<0.001	
pSWE (bilateral)	1.30 (1.2, 1.45)	1.71 (1.59, 1.85)	<0.001	<0.001

a Adjusted by age (adjustments are performed only for bilateral measurements as more reflective of the patients’ global features). GSA: grey-scale analysis; IQR: interquartile range; pSWE: point shear-wave elastography; SSc: systemic sclerosis.

The median (IQR) GSA value in the Jesi cohort was 93.3 (86.7–100.9), while in the Leeds cohort it was 95 (73.7–120.5). The median (IQR) pSWE value in the Jesi cohort was 1.3 (1.2–1.48), whereas the median (IQR) 2D SWE value in the Leeds cohort was 1.84 (1.66–1.97).

As illustrated in Table 4, in SSc patients, QM thickness was positively associated with muscle strength (i.e. HG test), better physical performance (i.e. SPPB) and lower disability (i.e. HAQ). Conversely, increased muscle echogenicity (both mHS and GSA) was associated with reduced muscle strength, poorer physical performance and greater disability (GSA only). Finally, higher pSWE values (Jesi cohort) were positively correlated with greater muscle strength, better physical performance and lower disability. In contrast, higher 2D SWE values (Leeds cohort) were correlated with reduced muscle strength and showed no significant association with disability.

**Table 4.** Correlation between different US modalities of muscle assessment (mass, quality and stiffness) and measures of disability, strength and physical performance in SSc patients

		Handgrip	**SPPB** ^a^	HAQ
Bilateral				
QM	rho	0.503	0.463	−0.257
	*P*-value	<0.001	0.004	0.031
mHS	rho	−0.489	−0.614	0.202
	*P*-value	<0.001	<0.001	0.096
GSA (Jesi)	rho	−0.57	−0.527	0.294
	*P*-value	<0.001	0.001	0.02
GSA (Leeds)	rho	−0.283	—	0.233
	*P*-value	0.001	—	0.032
pSWE (Jesi)	rho	0.481	0.572	−0.448
	*P*-value	0.003	<0.001	0.006
2D SWE (Leeds)	rho	−0.410	—	−0.150
	*P*-value	0.001	—	0.411
Left				
QM	rho	0.352	0.327	−0.249
	*P*-value	<0.001	0.05	0.0378
mHS	rho	−0.394	−0.507	0.173
	*P*-value	<0.001	0.02	0.155
GSA (Jesi)	rho	−0.504	−0.563	0.318
	*P*-value	<0.001	<0.001	0.03
GSA (Leeds)	rho	−0.267	—	0.174
	*P*-value	0.001	—	0.04
pSWE (Jesi)	rho	0.492	0.522	−0.463
	*P*-value	0.003	0.001	0.004
2D SWE (Leeds)	rho	−0.350	—	−0.164
	*P*-value	0.006	—	0.369
Right				
QM	rho	0.446	0.490	−0.199
	*P*-value	<0.001	0.002	0.098
mHS	rho	−0.473	−0.627	0.188
	*P*-value	<0.001	<0.001	0.121
GSA (Jesi)	rho	−0.507	−0.464	0.294
	*P*-value	<0.001	0.004	0.082
GSA (Leeds)	rho	−0.293	—	0.122
	*P*-value	<0.001	—	0.106
pSWE (Jesi)	rho	0.442	0.549	−0.387
	*P*-value	0.007	<0.001	0.019
2D SWE (Leeds)	rho	−0.407	—	−0.024
	*P*-value	0.001	—	0.897

a Measured in the Jesi cohort. 2D SWE: 2D shear-wave elastography; GSA: grey-scale analysis; HAQ: health assessment questionnaire; mHS: modified Heckmatt scale; pSWE: point shear-wave elastography; QM: quadriceps muscle; SSc: systemic sclerosis; SPPB: short physical performance battery; US: ultrasound.

As showed in Table 5, QM thickness was inversely correlated with muscle echogenicity (both mHS and GSA). Both muscle echogenicity measures displayed a good correlation between each other. In the Jesi cohort, pSWE was positively associated with QM thickness and inversely correlated with muscle echogenicity (both mHS and GSA). In the Leeds cohort, 2D SWE was inversely correlated with QM thickness with no significant correlation with muscle echogenicity.

**Table 5.** Correlation between the different US modalities of muscle assessment (muscle mass, muscle quality and muscle stiffness) in SSc patients

		GSA (Jesi)	GSA (Leeds)	mHS	pSWE (Jesi)	2D SWE (Leeds)
Bilateral						
QM thickness	rho	−0.607	−0.11	−0.35	0.608	−0.493
	*P*-value	<0.001	0.016	<0.001	<0.001	0.001
mHS	rho	0.727	0.487	—	−0.588	0.007
	*P*-value	<0.001	0.001	—	<0.001	0.964
GSA (Jesi)	rho	—	—	0.727	−0.641	—
	*P*-value	—	—	<0.001	<0.001	—
GSA (Leeds)	rho	—	—	0.487	—	−0.134
	*P*-value	—	—	0.001	—	0.398
Left						
QM thickness	rho	−0.573	−0.135	−0.28	0.494	−0.465
	*P*-value	0.001	0.03	<0.001	0.002	0.002
mHS	rho	0.657	0.555	—	−0.518	−0.110
	*P*-value	<0.001	<0.001	—	0.003	0.486
GSA (Jesi)	rho	—	—	0.657	−0.482	—
	*P*-value	—	—	<0.001	0.001	—
GSA (Leeds)	rho	—	—	0.555	—	0.046
	*P*-value	—	—	<0.001	—	0.771
Right						
QM thickness	rho	−0.563	−0.116	−0.37	0.594	−0.422
	*P*-value	<0.001	0.018	<0.001	<0.001	0.005
mHS	rho	0.709	0.421	—	−0.558	−0.017
	*P*-value	<0.001	0.005	—	<0.001	0.918
GSA (Jesi)	rho	—	—	0.709	−0.713	—
	*P*-value	—	—	<0.001	<0.001	—
GSA (Leeds)	rho	—	—	0.421	—	−0.016
	*P*-value	—	—	0.005	—	0.922

2D SWE: 2D shear-wave elastography; GSA: grey-scale analysis; mHS: modified Heckmatt scale; pSWE: point shear-wave elastography; QM: quadriceps muscle; SSc: systemic sclerosis; US: ultrasound.

As illustrated in Supplementary Table S3, in SSc patients, male sex was significantly associated with QM thickness and GSA (Jesi cohort). Additionally, QM thickness was positively correlated with ATA-positivity and negatively with GIT involvement. Lastly, DUs were negatively correlated with pSWE (Jesi cohort). No other significant correlations were found between US measurements and other SSc patients’ clinical features.

## Discussion

To our knowledge, this is the first study to assess muscle mass, quality and stiffness using US in SSc patients while examining their clinical relevance. Except for one study evaluating SWE [31], previous US studies have focused solely on muscle mass in SSc [32, 33]. Here, we introduce a novel multimodal US approach for evaluating sarcopenia-related muscle involvement in the largest SSc cohort studied to date.

Compared with HCs, SSc patients showed reduced QM thickness (indicative of lower muscle mass), though this was not significant after adjusting for age, and significantly higher echogenicity (reflecting poorer muscle quality). In the Jesi cohort—the only one with SWE data for HCs—SSc patients had lower pSWE values, suggesting reduced muscle stiffness. These US alterations were associated with decreased strength, worse physical performance and greater disability. Importantly, associations with reduced muscle mass and quality were consistent across both cohorts, reinforcing the role of US in the early detection of sarcopenia related muscle involvement.

Our cohort showed a low prevalence of probable sarcopenia (18.5%) based on HG strength per EWGSOP2 criteria [8], and few had severe SPPB impairment, indicating a ‘pre-sarcopenic’ phenotype. Disease severity was also mild, with limited organ involvement and low mRSS scores. Despite this, US identified early muscle changes that were clinically meaningful due to their association with muscle functional decline (i.e. reduced muscle strength and function).

Muscle mass remains a core imaging marker for sarcopenia [34]. A previous study by de Carvalho *et al.* reported a correlation between muscle mass and HG strength in 16 SSc patients [33]. In our cohort, after age adjustment, muscle mass in SSc patients was comparable to HCs. Notably, muscle mass was significantly associated with male sex, suggesting demographic factors may influence this parameter, especially in early or mild disease where muscle wasting is less evident.

Conversely, muscle quality was significantly impaired in SSc patients, as indicated by increased echogenicity—a marker of fat infiltration, with possible contributions from fibrosis and inflammatory change [32, 35, 36]. Previous studies suggest muscle quality often declines before mass loss, even in healthy individuals [3, 37]. Our findings imply that echogenicity changes may represent an early sign of muscle involvement in SSc. While our cross-sectional design limits causal inference, US may detect muscle quality alterations preceding mass reduction.

Regarding muscle stiffness, in the Jesi cohort, lower pSWE values correlated with reduced strength and performance, consistent with findings in SLE [13]. In contrast, the Leeds cohort showed higher 2D SWE values associated with reduced strength but not disability—potentially reflecting methodological differences. While pSWE in Jesi showed a strong positive correlation with QM thickness (suggesting that greater muscle mass is associated with greater stiffness), 2D SWE in Leeds showed a moderate negative correlation with QM and GSA. These discrepancies may reflect inherent differences between SWE methods, as noted in liver studies [29, 30]. 2D SWE samples larger areas, possibly capturing composite features like fibrosis, fat infiltration, oedema, and atrophy [38, 39]. Other influencing factors include anisotropy, contraction state, tissue heterogeneity, and technical aspects like probe placement and pressure [40, 41]. Additionally, SSc-specific factors such as subclinical fibrosis, microvascular changes, or low-grade inflammation may contribute to variable muscle stiffness, potentially leading to increased stiffness in the presence of fibrotic infiltration, or decreased stiffness in cases of muscle fibre loss, oedema or early atrophic changes. However, as each patient was scanned using only one US system and histopathology was unavailable, we cannot definitively differentiate between technical and biological sources of variability. Our findings highlight the need for standardization and further research to clarify the clinical applications of SWE, particularly when using different techniques, such as pSWE and 2D SWE, in muscle assessment.

Our study demonstrates that combining muscle mass measurement, echogenicity analysis, and stiffness assessment using SWE—a ‘multimodal US’ approach—offers a comprehensive evaluation of muscle pathology in SSc patients. Early detection of muscle abnormalities could enable timely interventions, such as exercise, medications, or supplements, to slow deterioration. These proactive strategies may help preserve strength and function, reducing long-term complications like disability and diminished quality of life. Farrow *et al.* reported reduced skeletal muscle mass and strength in treatment-naïve individuals with newly diagnosed rheumatoid arthritis, compared with age-matched HCs [42]. These impairments persisted even during long-standing clinical remission, highlighting that early, possibly irreversible muscle degeneration can occur in inflammatory diseases—underscoring the importance of early identification and intervention.

Our findings also suggest systemic associations between muscle US findings and SSc manifestations. Specifically, we observed links between reduced muscle mass and GIT involvement, and between pSWE and DUs. Vascular damage, reflected by DUs, may contribute to GI dysfunction and malnutrition, thereby impacting muscle health. This supports the potential of muscle US to serve as a surrogate marker for identifying patients at higher risk of sarcopenia, guiding preventive strategies such as nutritional and rehabilitative interventions [43, 44]. However, given the cross-sectional design, these findings remain associative, and longitudinal studies are needed to explore underlying mechanisms.

We selected the QM for US evaluation due to its high reliability and strong correlation with reference imaging methods (MRI, CT and DXA) used to assess appendicular lean mass [45–47]. The QM is also among the earliest muscles to show age-related atrophy [48], and its size and accessibility make it a frequent target in both clinical and research settings. Moreover, the multimodal US protocol used here—focused on the QM—has shown excellent inter- and intra-rater reliability in previous web-based and patient-based validation studies [49, 50], supporting its applicability in SSc and broader rheumatic populations.

This study’s key strength lies in its novelty, offering the first detailed multimodal US assessment of muscle pathology in SSc patients. It evaluates muscle mass, quality and stiffness while exploring correlations with strength, function and clinical features. A large, multicentric cohort enhances statistical power, reduces bias and improves generalizability. The use of a validated US protocol ensures reliability, highlighting US as a promising tool for muscle assessment in research and clinical practice.

The main limitation of this study is its cross-sectional design, which prevents evaluation of muscle progression and treatment response. Longitudinal studies are needed to determine whether early US-detected abnormalities predict outcomes such as disability, functional decline and treatment response in SSc patients. Another limitation is the absence of a control group in the Leeds cohort, precluding direct GSA and 2D SWE comparisons with HCs. The older age of the SSc cohort may also have influenced muscle measurements. Although age adjustments were applied, future studies with matched controls are needed to separate disease-specific changes from age-related ones.

Functional assessments varied by site: the SPPB was used only in Jesi, which differed clinically from Leeds. Nonetheless, key US parameters correlated with function in both cohorts, supporting their clinical relevance. While patients with overt myopathy or malnutrition were excluded to isolate sarcopenia-related changes, this may introduce an artificial dichotomy. Subclinical inflammation, fibrosis and other SSc-related factors may still affect muscle pathology. Including a broader SSc spectrum in future research could help identify early changes and their role in sarcopenia.

Furthermore, HG strength may have been influenced by arthritis, sclerodactyly or tendinopathy. Although arthritis and sclerodactyly were recorded, tendinopathy was not systematically assessed and represents an additional limitation. Lower limb performance measures such as walking speed would have been informative but were not feasible in both cohorts. While SPPB offered indirect insight in Jesi, HG strength remains a practical proxy for global muscle function in clinical settings.

Moreover, the lack of a gold standard imaging method (e.g. MRI or DXA) limits US validation. Future studies should compare US findings with these modalities. Lastly, although the US protocol was previously validated [49, 50], it was not re-tested with the new operator. However, the protocol was reviewed and tested before enrolment to minimize inter-operator variability.

## Conclusions

This study provides new insights into muscle mass, quality, stiffness, and their relationships with muscle strength, function and disability in SSc patients. US can detect early clinically relevant muscle involvement, potentially enabling timely interventions to improve patient outcomes.

Our findings suggest that alterations in muscle quality may represent a sensitive marker of clinically relevant muscle involvement in SSc, whereas muscle mass—though correlated with physical performance measures—is more strongly influenced by factors such as age and sex. Future research should further clarify the role of SWE in muscle assessment, particularly by comparing pSWE and 2D SWE techniques.

## Supplementary Material

keaf415_Supplementary_Data

## Data Availability

The data are available from the corresponding author upon reasonable request.
