# Supplementary material for: Multimodal ultrasound in the assessment of muscle involvement in systemic sclerosis patients: results from a bicentric study
Source: Rheumatology (Oxford). 2025 Aug 6;64(12):6159–67. doi: 10.1093/rheumatology/keaf415 (PMC12671856; doi:10.1093/rheumatology/keaf415)
Supplement: keaf415_Supplementary_Data [file keaf415_supplementary_data.docx]

**Supplementary Table S1:** Clinical characteristics of SSc patients.

| **SSc disease-specific characteristics** | |
| --- | --- |
| **mRSS, median (IQR)** | 3.0 (0.0, 8.0) |
| **Leroy subset** |  |
| Diffuse cutaneous (%) | 19 (23.5) |
| Limited cutaneous (%) | 62 (76.5) |
| **Autoantibodies** |  |
| ATA (%) | 19 (23.5) |
| ACA (%) | 38 (47.0) |
| ARA (%) | 4 (4.9) |
| PM/Scl (%) | 5 (6.2) |
| **NVC** |  |
| Non-specific/Normal (%) | 13 (16.0) |
| Early SSc (%) | 21 (25.9) |
| Active SSc (%) | 26 (32.1) |
| Late SSc (%) | 12 (14.8) |
| **Organ manifestations** |  |
| RP (%) | 80 (98.8) |
| DU (%) | 28 (34.6) |
| Sclerodactyly (%) | 19 (23.5) |
| ILD (%) | 28 (34.6) |
| PAH (%) | 9 (11.1) |
| Joint (%) | 6 (7.4) |
| Heart (%) | 7 (8.6) |
| Respiratory (%) | 33 (40.7) |
| GI (%) | 46 (56.8) |
| Borg scale, median (IQR) | 2 (0, 3) |
| **Treatments** |  |
| Mycophenolate Mofetil (%) | 23 (28.4) |
| Hydroxychloroquine (%) | 15 (18.5) |
| Methotrexate (%) | 7 (8.6) |
| Rituximab (%) | 7 (8.6) |
| Glucocorticoid use (%) | 4 (4.9) |
| ERA (%) | 20 (24.7) |
| Iloprost (%) | 13 (16.0) |
| CCB (%) | 33 (40.7) |
| PDE5i (%) | 24 (29.6) |
| **Comorbidities** |  |
| Cardiovascular (%) | 15 (18.5) |
| Metabolic disorders (%) | 14 (17.3) |
| Gastrointestinal (%) | 12 (14.8) |
| Malignancies (%) | 6 (7.4) |
| Respiratory (%) | 3 (3.7) |

Acronyms. ACA = anti-centromere antibody, ARA = anti-RNA polymerase III antibody, ATA = anti-topoisomerase antibody, CCB = calcium channel blocker, DU = digital ulcer, ERA = endothelin receptor antagonist, GI = gastrointestinal involvement, ILD = interstitial lung disease, mRSS = modified Rodnan Skin Score, PAH = pulmonary artery hypertension, PM/Scl = Anti-Polymyositis /Scleroderma Antibody, RP = Raynaud’s phenomenon, SSc = systemic sclerosis.

**Supplementary Table S2.** Comparison of clinical and demographic characteristics between SSc patients across the Jesi and Leeds cohorts

| **Characteristic** | **Jesi**  **(N=36)** | **Leeds**  **(N=45)** | **p-value** |
| --- | --- | --- | --- |
| Age, median (IQR), years | 55.5 (51.3, 66.5) | 59 (51, 67) | 0.615 |
| Male sex (%) | 3 (8.3) | 8 (17.7) | 0.183 |
| Disease duration in years, median (IQR) | 6 (3.3, 10) | 7 (4.2, 12) | 0.269 |
| BMI, median (IQR) | 24.8 (21.1, 28.7) | 24.6 (22.3, 30.1) | 0.791 |
| Smokers (%) | 3 (8.3) | 3 (6.7) | 0.598 |
| HAQ, median (IQR) | 0.38 (0.1, 0.8) | 0.8 (0.3, 1.8) | 0.016 |
| Handgrip test, mean ± SD, kg | 29 ± 8.4 | 25 ± 10 | 0.02 |
| **Disease-specific characteristics** |  |  |  |
| **mRSS, median (IQR)** | 6 (0, 10) | 1 (0; 4.5) | 0.01 |
| **Pattern** |  |  |  |
| Diffuse (%) | 6 (16.7) | 13 (28.9) | 0.127 |
| Limited (%) | 30 (83.3) | 32 (71.1) |  |
| **Autoantibodies** |  |  |  |
| ATA (%) | 13 (36.1) | 6 (13.3) | 0.016 |
| ACA (%) | 19 (52.8) | 19 (42.2) | 0.235 |
| ARA (%) | 1 (2.8) | 3 (6.7) | 0.397 |
| PM/Scl (%) | 0 (0) | 5 (11.1) | 0.048 |
| **Organ manifestations** |  |  |  |
| RP (%) | 36 (100) | 44 (97.8) | 0.556 |
| DU (%) | 8 (22.2) | 20 (44.4) | 0.014 |
| Sclerodactyly | 8 (22.2) | 11 (24.4) | 0.815 |
| ILD (%) | 16 (44.4) | 12 (26.7) | 0.452 |
| PAH (%) | 4 (11.1) | 4 (8.9) | 0.637 |
| Joint (%) | 1 (2.8) | 5 (11.1) | 0.069 |
| Heart (%) | 5 (13.9) | 2 (4.4) | 0.157 |
| GIT (%) | 14 (38.9) | 32 (71.1) | 0.01 |
| Borg Scale, median (IQR) | 3 (0.6, 3.8) | 0.5 (0, 2.8) | 0.03 |
| **Treatments** |  |  |  |
| Mycophenolate Mofetil (%) | 16 (44.4) | 7 (15.6) | 0.088 |
| Hydroxychloroquine (%) | 5 (13.9) | 10 (22.2) | 0.253 |
| Methotrexate (%) | 2 (5.6) | 5 (11.1) | 0.319 |
| Rituximab (%) | 1 (2.8) | 6 (13.3) | 0.097 |
| Glucocorticoid use (%) | 1 (2.8) | 3 (6.7) | 0.397 |
| ERA (%) | 12 (33.3) | 8 (17.7) | 0.03 |
| Iloprost (%) | 6 (16.7) | 7 (15.6) | 0.564 |
| CCB (%) | 16 (44.4) | 17 (37.7) | 0.352 |
| PDE5i (%) | 6 (16.7) | 18 (0.4) | 0.019 |

Acronyms. ACA = anti-centromere antibody, ARA = anti-RNA polymerase III antibody, ATA = anti-topoisomerase antibody, BMI = body mass index, CCB = calcium channel blocker, DU = digital ulcer, ERA = endothelin receptor antagonist, GI = gastrointestinal involvement, HAQ = health assessment questionnaire, ILD = interstitial lung disease, mRSS = modified Rodnan Skin Score, PAH = pulmonary artery hypertension, PM/Scl = Anti-Polymyositis /Scleroderma Antibody, RP = Raynaud’s phenomenon, SSc = systemic sclerosis.

**Supplementary Table S3.** Correlation between the different US measurements and demographic and clinical characteristics of SSc patients

|  | **Correlation strength** | **QM** | **GSA**  **(Jesi)** | **GSA**  **(Leeds)** | **mHS** | **pSWE (Jesi)** | **2D SWE (Leeds)** |
| --- | --- | --- | --- | --- | --- | --- | --- |
| **Age** | rho | -0.431 | 0.502 | 0.076 | 0.157 | -0.548 | 0.141 |
|  | p-value | p=0.000 | p=0.002 | p=0.043 | p=0.170 | p=0.001 | p=0.378 |
| **BMI** | rho | 0.258 | -0.390 | -0.079 | -0.101 | -0.104 | -0.380 |
|  | p-value | p=0.027 | p=0.19 | p=0.143 | p=0.396 | p=0.545 | p=0.022 |
| **Male sex** | rho | 0.228 | -0.436 | -0.063 | -0.215 | 0.232 | -0.354 |
|  | p-value | p=0.041 | p=0.08 | p=0.312 | p=0.055 | p=0.172 | p=0.02 |
| **Disease duration** | rho | -0.205 | -0.085 | -0.044 | 0.003 | -0.090 | 0.247 |
|  | p-value | p=0.074 | p=0.623 | p=0.786 | p=0.977 | p=0.601 | p=0.130 |
| **Borg scale** | rho | 0.084 | -0.002 | 0.173 | 0.094 | -0.185 | 0.045 |
|  | p-value | p=0.497 | p=0.991 | p=0.352 | p=0.450 | p=0.280 | p=0.808 |
| **ATA positivity** | rho | 0.284 | -0.053 | -0.076 | -0.11 | 0.178 | -0.011 |
|  | p-value | p=0.01 | p=0.759 | p=0.626 | p=0.333 | p=0.298 | p=0.945 |
| **CENP-positivity** | rho | -0.146 | 0.059 | 0.16 | 0.243 | -0.282 | -0.126 |
|  | p-value | p=0.193 | p=0.733 | p=0.299 | p=0.03 | p=0.096 | p=0.419 |
| **Diffuse subset** | rho | -0.004 | 0.147 | -0.25 | -0.152 | -0.07 | -0.197 |
|  | p-value | p=0.973 | p=0.392 | p=0.873 | p=0.184 | p=0.967 | p=0.217 |
| **DU** | rho | -0.184 | 0.039 | -0.113 | 0.104 | -0.132 | 0.271 |
|  | p-value | p=0.106 | p=0.823 | p=0.489 | p=0.184 | p=0.0443 | p=0.95 |
| **ILD** | rho | 0.059 | 0.04 | -0.061 | -0.142 | 0.02 | -0.161 |
|  | p-value | p=0.604 | p=0.818 | p=0.703 | p=0.214 | p=0.908 | p=0.316 |
| **mRSS** | rho | 0.096 | 0.272 | -0.06 | 0.097 | -0.118 | 0.138 |
|  | p-value | p=0.405 | p=0.109 | p=0.971 | p=0.403 | p=0.492 | p=0.402 |
| **GI**  **involvement** | rho | -0.242 | -0.05 | -0.016 | -0.105 | 0.025 | 0.190 |
|  | p-value | p=0.032 | p=0.975 | p=0.922 | p=0.362 | p=0.886 | p=0.235 |
| **VCS pattern** | rho | 0.064 | 0.245 | 0.04 | 0.01 | -0.148 | 0.2 |
|  | p-value | p=0.592 | p=0.149 | p=0.98 | p=0.932 | p=0.388 | p=0.248 |

Acronyms. 2D SWE = 2D shear wave elastosonography, ACA = anti-centromere antibody, ATA = anti-topoisomerase I antibody, BMI = body mass index, CENP = anti-centromere protein antibody, DU = digital ulcer, GI = gastrointestinal involvement, GSA = grey-scale analysis, ILD = interstitial lung disease, mHS = modified Heckmatt scale, mRSS = modified Rodnan skin score, pSWE = point shear wave elastosonography, QM = quadriceps muscle thickness SSc = systemic sclerosis, US = ultrasound, VCS = videocapillaroscopy.

**Supplementary Figure S1.** Representative ultrasound images of the quadriceps muscle in patients with systemic sclerosis.


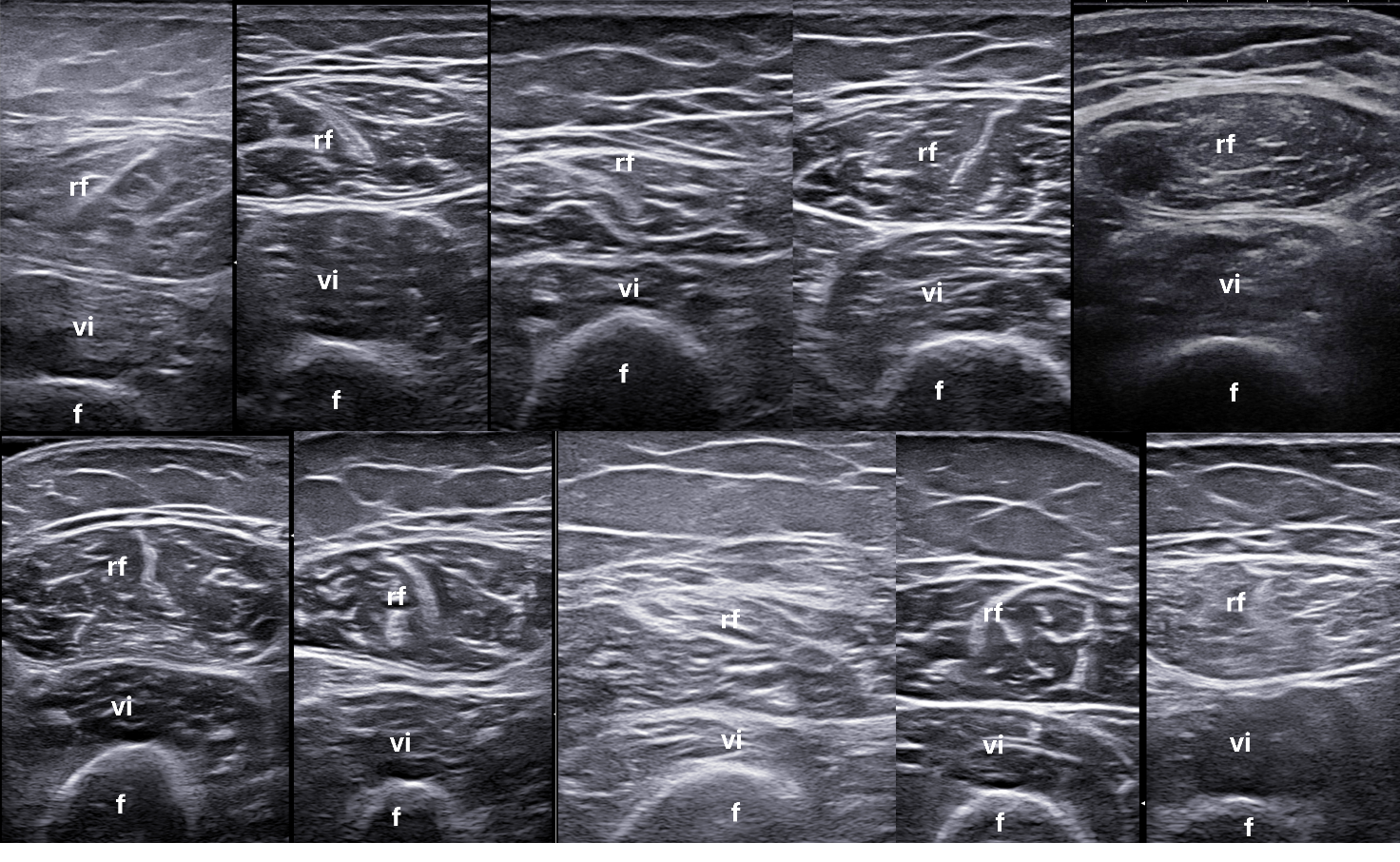

Images illustrate varying degrees of increased muscle echogenicity and altered muscle architecture in SSc patients. These qualitative differences suggest early or subclinical muscle involvement that may not be accompanied by overt muscle atrophy. Acronyms: f: femur, rf: rectus femoris muscle, vi: vastus intermedius muscle.
